# Association Between Components of Cognitive Reserve and Serum BDNF in Healthy Older Adults

**DOI:** 10.3389/fnagi.2021.725914

**Published:** 2021-08-02

**Authors:** Jessica M. Collins, Edward Hill, Aidan Bindoff, Anna E. King, Jane Alty, Mathew J. Summers, James C. Vickers

**Affiliations:** ^1^Wicking Dementia Research and Education Centre, University of Tasmania, Hobart, TAS, Australia; ^2^School of Health and Behavioral Sciences, University of the Sunshine Coast, Sippy Downs, QLD, Australia

**Keywords:** education, serum, polymorphism, cognitive reserve, brain derived neurotrophic factor, Alzheimer’s disease, biomarkers

## Abstract

**Background**: The brain-derived neurotrophic factor (BDNF) protein has been shown to have a prominent role in neuron survival, growth, and function in experimental models, and the BDNF Val66Met polymorphism which regulates its expression has been linked to resilience toward the effects of aging on cognition. Cognitively stimulating activity is linked to both increased levels of BDNF in the brain, and protection against age-related cognitive decline. The aim of this study was to investigate the associations between serum BDNF levels, the BDNF Val66Met genotype, and components of cognitive reserve in early and mid-life, measured with the Lifetime of Experiences Questionnaire (LEQ).

**Methods**: Serum BDNF levels were measured cross-sectionally in 156 participants from the Tasmanian Healthy Brain Project (THBP) cohort, a study examining the potential benefits of older adults engaging in a university-level education intervention. Multiple linear regression was used to estimate serum BDNF’s association with age, education, gender, *BDNF* Val66Met genotype, later-life university-level study, and cognitively stimulating activities measured by the LEQ.

**Results**: Serum BDNF in older adults was associated with early life education and training, increasing 0.007 log(pg/ml) [95%CI 0.001, 0.012] per unit on the LEQ subscale. Conversely, education and training in mid-life were associated with a −0.007 log(pg/ml) [−0.012, −0.001] decrease per unit on the LEQ subscale. Serum BDNF decreased with age (−0.008 log(pg/ml) [−0.015, −0.001] per year), and male gender (−0.109 log(pg/ml) [−0.203, −0.015]), but mean differences between the *BDNF* Val66Met polymorphisms were not significant (*p* = 0.066). All effect sizes were small, with mid-life education and training having the largest effect size ($\eta_{p}^{2}$ = 0.044).

**Conclusion**: Education in both early and mid-life explained small but significant amounts of variance in serum BDNF levels, more than age or gender. These effects were opposed and independent, suggesting that education at different stages of life may be associated with different cognitive and neural demands. Education at different stages of life may be important covariates when estimating associations between other exposures and serum BDNF.

## Introduction

A of evidence suggests an individual’s risk of developing dementia is influenced by genetic and environmental factors, and their interactions (Lourida et al., 2019). One environmental factor which has been strongly linked to dementia risk is early life education, with lower levels of education conferring an increased risk of dementia (Livingston et al., 2020) and cognitive stimulation suggested to improve resilience to dementia. This evidence, in combination with post-mortem studies demonstrating some people without clinical symptoms of dementia prior to death, exhibit high levels of dementia-related pathology in the brain (Roe et al., 2007; Price et al., 2009), has led to the proposition of a theoretical construct known as “cognitive reserve.” The cognitive reserve can thus be conceptualized as an individual’s resilience to neuropathological insult.

Although the mechanisms underlying cognitive reserve are unclear, the links between resilience to brain pathology and cognitive stimulation (Stern, 2002) suggest a dependence on synaptic plasticity. The protein, brain-derived neurotrophic factor (BDNF) has been implicated in the beneficial effects of cognitively stimulating activities as it is a regulator of synaptic transmission, inducing long-term changes in excitability and synaptic plasticity in the adult brain (Miranda et al., 2019). Cognitive stimulation, which increases BDNF levels in the brain in BDNF+/− mice compared with wildtype (Novkovic et al., 2015), has also been shown to play an important role in learning and memory (Mather, 2020). Higher serum BDNF levels have been suggested to protect against dementia occurrence (Weinstein et al., 2014), therefore they may represent an important mediator of the beneficial effects of cognitively stimulating activities on cognitive reserve and dementia risk.

The BDNF gene has a common single nucleotide polymorphism causing a valine to methionine amino acid substitution at residue 66 (Val66Met) and Met allele carriers have been shown to exhibit reduced synaptic activity and greater cognitive decline compared to Val/Val homozygotes (Miyajima et al., 2008; Lim et al., 2013). In healthy adults aged 71.36 (±7.15) years with evidence of amyloid pathology, Met carriers showed significant declines in episodic memory, executive function, and language compared to their Val/Val homozygote counterparts (Lim et al., 2013). However, other studies have shown conflicting results and meta-analyses have failed to establish an association with cognition (Mandelman and Grigorenko, 2012). Therefore, the effect of the BDNF Val66Met polymorphism on healthy cognitive ability requires further investigation, particularly the relationship between the BDNF Val66Met polymorphism, cognitive stimulation, and cognitive reserve.

The expression of BDNF is tightly regulated and its synthesis and secretion by neurons is affected by multiple factors including stress, exercise, and learning (Ickes et al., 2000; Mattson et al., 2004). Furthermore, BDNF released in the brain, can cross the blood-brain barrier (Pan et al., 1998) and is present in peripheral circulating blood in both plasma and platelets (Radka et al., 1996). Serum BDNF levels are more than 100 times higher than plasma BDNF levels. This is due to the degranulation of platelets during the clotting process, which contain high amounts of protein (Rosenfeld et al., 1995; Radka et al., 1996; Fujimura et al., 2002). In peripheral blood, platelets circulate for 11 days, whereas the BDNF protein circulates in plasma for less than 1 h. Thus, serum BDNF levels may represent a long-term measure of brain BDNF levels. In contrast, plasma BDNF concentrations may vary with events in the hours leading up to blood collection (Lommatzsch et al., 2005).

A study in rats indicates that BDNF levels in both plasma and serum correlate with brain levels of the protein (Karege et al., 2002), and several non-human studies have described circulating levels of BDNF as a proxy for expression in the brain (Sartorius et al., 2009; Klein et al., 2011). Human studies of BDNF levels in serum and the brain have demonstrated levels decrease with age and that lower serum BDNF levels are associated with lower spatial memory performance (Erickson et al., 2010). Furthermore, a community-based prospective study of 2,131 participants, followed for up to 10 years by Weinstein et al. (2014), found that higher serum BDNF levels were associated with a reduction in risk of dementia and Alzheimer’s disease (AD). However, studies examining the effect of the BDNF Val66Met polymorphism on brain and serum BDNF levels demonstrate mixed results, with Met carriers reported to have both lower (Ozan et al., 2010), higher (Lang et al., 2009; Minelli et al., 2011) and similar (Yu et al., 2008) levels compared to Val homozygotes. Serum BDNF levels also display heterogeneity with respect to gender (Lang et al., 2009; Ozan et al., 2010; Shimada et al., 2014), therefore the relationship between circulating BDNF levels, gender, BDNF genotype, and age-related cognitive decline is unclear.

The Tasmanian Healthy Brain Project (THBP) is a prospective longitudinal study of cognitively healthy people aged 50 years or more with a self-nominated intervention group undertaking later life university-level education (Summers et al., 2013). The overall aim of the project is to investigate the potential of later-life university-level education to increase cognitive performance, enhance cognitive reserve, and subsequently reduce age-related cognitive decline. Previously, data from this study has demonstrated that the BDNF Val66Met polymorphism positively moderates the relationship between cognitive reserve and executive function in Val homozygotes, but not in Met carriers (Ward et al., 2017). It has also shown that later-life university-level education leads to a significant increase in cognitive reserve (Lenehan et al., 2016) and language processing capacity (Thow et al., 2018). Additionally, the amount of education undertaken benefited language processing performance in a dose-dependent manner in BDNF Met carriers, but not Val homozygotes (Ward et al., 2019). Using cross-sectional data from the THBP, the current exploratory study aimed to determine whether BDNF levels in serum were related to the BDNF Val66Met genotype. This study was conducted in non-impaired cognitively healthy adults, which is particularly important given the pre-clinical prodrome in cognitive decline, and the necessity to conduct preventative research as early as possible along the disease course. Furthermore, we aimed to determine whether serum BDNF levels were related to the participants’ cognitive reserve, using subscales of the Lifetime of Experiences Questionnaire (LEQ) to measure education and training, career attainment, and other cognitively stimulating activities in young adulthood and mid-life. Finally, we were interested to investigate how later-life university study, the hallmark intervention of the THBP, related to serum BDNF levels in healthy older adults.

## Materials and Methods

### Cognitive Reserve

The THBP is an ongoing longitudinal study that commenced in 2011. For the first 4 years, participants attend annual clinical assessments, followed by biennial, full details have been published previously (Summers et al., 2013; Ward et al., 2014). Assessments are conducted in-person at a clinical research facility in Hobart, Tasmania, by a trained neuropsychologist. At each assessment, participants complete a medical health status questionnaire assessing health, medical conditions, prescription medication use, and drug and alcohol use for the preceding 12/24 months. This questionnaire was designed specifically for the THBP, collecting demographic information such as age (in years), gender (options either “male” or “female”), handedness, height (in cm), weight (in kg), marital status, education (in years) and occupational history.

To account for THBP participant’s engagement with cognitively stimulating activities at different stages across the lifespan, the cognitive reserve was treated as individual subscales from; university study (participation in later-life education as part of the THBP), premorbid intelligence quotient [Weschler Test of Adult Reading (WTAR), Full-Scale IQ (FSIQ)], and lifetime cognitive stimulation [Lifetime Experiences Questionnaire (LEQ)]. Both the WTAR and LEQ were completed at baseline, whilst participation in the THBP educational intervention was calculated retrospectively. Developed by Valenzuela and & Sachdev (2007), the LEQ assesses educational, occupational, and cognitive lifestyle activities at different stages through life. We used the LEQ’s early life and midlife indicators of cognitive reserve; young adult specific, young adult nonspecific, midlife specific, and midlife bonus. A score on the LEQ young adulthood specific of 15.7 could represent the completion of approximately 5 years of secondary school and a university undergraduate degree, whilst a score of 10.1 for midlife bonus could represent the completion of a Ph.D. We did not assess the later-life subscales of the LEQ because few participants were old enough to complete this section. WTAR-FSIQ provides a stable and reliable estimate of premorbid intellectual capacity (The Psychological Corporation, 2001). For the WTAR-FSIQ, participants were given the word list at neuropsychological assessment and asked to articulate 50 words with an atypical grapheme to phoneme translation (such as “ballet” or “aisle”). Finally, a participant’s engagement with the THBP university-level education was included, measured as years of full-time study equivalent at university completed as part of the THBP intervention.

### Genotyping

Participants of the THBP were genotyped as previously described in 2014 (Stuart et al., 2014). DNA samples were extracted from saliva and collected with Oragene DNA self-collection kits (Genotek, ON, Canada, 2012). *BDNF* genotypes were determined through one-step amplified refractory mutation system PCR31 and subsequent gel electrophoresis. Genetic polymorphisms were determined by following previously described methods for *BDNF* (Sheikh et al., 2010). PCR amplifications were undertaken in a 12-μl reaction volume that contained ~50 ng of genomic DNA. PCR amplicons were resolved on 2% agarose gel.

### Blood Collection

Blood was collected, processed, and stored according to published guidelines for AD research (O’Bryant et al., 2015). Briefly, non-fasting blood was collected by venepuncture with aseptic technique using a 21-gauge (G) butterfly needle into BD Vacutainer^TM^ SST^TM^ II Advance tubes (Cat no. 367958). For the preparation of serum, blood was clotted in a vertical position at room temperature for 30 min prior to centrifugation at 1,500 *g* for 10 min at 4°C. Serum was then aliquoted into 10 polypropylene, screw-top cryostorage tubes to prevent multiple freeze-thaw cycles, and stored at −80°C. The time from venepuncture to the storage of serum and plasma samples at −80°C was under 2 h.

### BDNF

BDNF measurements in serum samples were performed using the BDNF Discovery Kit single-molecule array assay (Simoa^®^) from Quanterix (Cat no. 102039). Calibrators, participant samples, and two quality control samples per plate were measured in duplicate using a 2-step assay on a Simoa SR-X platform (Quanterix) according to manufactures protocols. Serum samples were diluted 1/1,000. The intra-assay concentration coefficient of variation was calculated for the duplicates of each participant sample and if they were more than 20% the sample was remeasured as per De Wolf et al. (2020). The intra-assay coefficient of variation for included sample measures was 7.2%. The median values for bi-participant replicates were then calculated and if the absolute difference between a replicate and the median value exceeded 10% of the median, the replicate was treated as an outlier and deleted from the statistical analysis. The mean of each biomarker concentration was then calculated for each participant sample.

### Participants

A total of 566 THBP participants were progressively recruited from 2011 to 2014 through general advertising including newspaper, website, radio, television, and public lectures. Participants were eligible if there was no history of prior conditions known to be associated with an impaired neurological function such as dementia; multiple sclerosis; prior head injury requiring hospitalization; epilepsy; cerebrovascular complications including stroke, aneurysm, transient ischaemic attacks; poorly controlled diabetes; poorly controlled hypertension or hypotension; other neurological disorders, e.g., cerebral palsy or spina bifida; chronic obstructive pulmonary disease; heart disease; partial or total blindness; deafness; current psychiatric diagnosis (for further details please see Summers et al., 2013). On entry into the THBP, and reflecting a lengthy “real-world” intervention, participants opted (non-random assignment) into university-level education (439 later-life university study, 78%) or no further education (127 control, 22%). Participation in the THBP is ongoing and participants are still completing university-level subjects as part of the educational intervention (Hill et al., 2020). To date, 156 participants (27.5% over 9 years) have withdrawn, with dropout proportions similar to group proportions (44 control, 28%; 112 intervention, 72%). Of the participants who withdrew, the majority cited factors unrelated to the study as their reason of withdrawal: 22% relocated, 13% unable to recontact, 9% too busy, 10% medical diagnosis, 6% deceased, 3% work commitments, 2% family issues, 28% provided no reason; and 7% reported the assessments too stressful. All THBP participants were invited to take part in this biomarker sub-study and blood samples were collected from 174 (39 control, 22%; 135 intervention, 78%) participants in 2018. All participants were asked to avoid exercise in the 24 h prior to their blood collection. This project received ethical approval from the University of Tasmania Health and Medical HREC (H0018265 and H0016317) and was carried out in accordance with the Declaration of Helsinki. All participants signed written informed consent before participation.

### Statistics

In order to determine which variables (including potential confounders) were associated with serum BDNF, a multiple linear regression model (Model 1) was fitted which included all LEQ young adulthood and mid-life subscales, age in years, self-reported gender, WTAR-FSIQ, *BDNF* Val66Met polymorphism, and years of full-time equivalent university study as part of the THBP. Terms in Model 1 with *p* < 0.10 were included in a final model (Model 2) for inference (Chowdhury and Turin, 2020; age was retained as a covariate on the basis of strong previous evidence demonstrating a relationship between age and serum BDNF levels e.g., Erickson et al., 2010; Shimada et al., 2014). Variables in Model 2 with *p* < 0.05 were considered significant. F-statistics were computed using type 2 sums of squares. Serum BDNF (pg/ml) was log_e_-transformed to improve the normality of residuals following inspection of Q-Q plots. We have reported partial eta-squared as a standardized measure of effect size since it is easily interpreted as the proportion of variance explained by a single variable in a model with many variables. All analysis was conducted in the R environment for statistical computing (R Core Team). Reproducible R code is provided at https://github.com/ABindoff/bdnf_cr.

## Results

### Descriptive Statistics

A total of 174 participants provided blood samples for analysis. After excluding outliers based on serum BDNF measurements (*n* = 18), samples from 156 participants were used in the analysis. Participants had an average age of 69.8 years (SD 6.2, range 58–84) and 14.1 years (SD 2.4) of early life education (Table 1). A total of 107 (69%) participants were female. Of the 145 participants with genotypic data (11 missing), 89 were *BDNF* Val66Met− (57.1%) while 56 were *BDNF* Val66Met+ (35.9%). Mean serum levels of BDNF were 20,600 pg/ml (SD 5760; Table 1). Serum BDNF levels were higher in females [21,100 (SD = 5,500) pg/ml] compared with males [19,700 (SD = 6,250) pg/ml].

**Table 1:** Characteristics of all included THBP participants (*n* = 156).

	Female (*N* = 107)	Male (*N* = 49)	Overall (*N* = 156)
**BDNF in serum log(pg/ml)**
Mean (SD)	21,100 (5500)	19,700 (6250)	20,600 (5760)
Median [Min, Max]	20,300 [9820, 41700]	18,500 [7810, 35000]	20,000 [7810, 41700]
**Age (years)**			
Mean (SD)	69.6 (6.39)	70.1 (5.90)	69.8 (6.22)
Median [Min, Max]	70.0 [58.0, 84.0]	71.0 [58.0, 83.0]	70.5 [58.0, 84.0]
**BDNF Val66Met genotype**			
met-	61.0 (57.0%)	28.0 (57.1%)	89.0 (57.1%)
met+	37.0 (34.6%)	19.0 (38.8%)	56.0 (35.9%)
Missing	9.00 (8.4%)	2.00 (4.1%)	11.0 (7.1%)
**Education (early life)**			
Mean (SD)	13.8 (2.42)	14.7 (2.33)	14.1 (2.42)
Median [Min, Max]	13.0 [9.00, 21.0]	15.0 [10.0, 19.0]	14.0 [9.00, 21.0]
**FTE Years of university study (THBP intervention)**			
Mean (SD)	1.26 (1.39)	1.00 (1.18)	1.18 (1.33)
Median [Min, Max]	0.875 [0, 7.50]	0.500 [0, 4.38]	0.750 [0, 7.50]
**LEQ young adulthood specific (education)**			
Mean (SD)	14.2 (7.31)	18.8 (7.68)	15.7 (7.70)
Median [Min, Max]	12.6 [3.15, 44.1]	18.9 [4.20, 35.7]	15.5 [3.15, 44.1]
**LEQ young adulthood general**			
Mean (SD)	24.2 (5.74)	25.6 (5.35)	24.6 (5.64)
Median [Min, Max]	24.0 [11.0, 38.0]	26.0 [15.0, 34.0]	24.0 [11.0, 38.0]
**LEQ midlife specific (occupation)**			
Mean (SD)	18.7 (4.72)	20.9 (3.30)	19.4 (4.43)
Median [Min, Max]	19.0 [2.50, 25.5]	21.0 [14.0, 25.5]	19.5 [2.50, 25.5]
**LEQ midlife general**			
Mean (SD)	24.8 (4.89)	25.0 (4.73)	24.9 (4.83)
Median [Min, Max]	25.0 [13.0, 36.0]	26.0 [11.0, 33.0]	25.0 [11.0, 36.0]
**LEQ midlife bonus (education)**			
Mean (SD)	9.79 (7.75)	9.10 (9.11)	9.57 (8.18)
Median [Min, Max]	8.40 [0, 32.6]	8.40 [0, 46.2]	8.40 [0, 46.2]
Missing	1.00 (0.9%)	0 (0%)	1.00 (0.6%)
**LEQ later-life specific (occupation)**			
Mean (SD)	7.95 (4.69)	6.72 (1.35)	7.56 (3.96)
Median [Min, Max]	7.38 [4.00, 33.0]	6.50 [4.25, 8.75]	7.00 [4.00, 33.0]
Missing	71.0 (66.4%)	32.0 (65.3%)	103 (66.0%)
**LEQ later-life general**			
Mean (SD)	23.9 (6.15)	21.0 (3.81)	23.0 (5.64)
Median [Min, Max]	24.5 [1.20, 38.0]	21.0 [16.0, 30.0]	23.0 [1.20, 38.0]
Missing	71.0 (66.4%)	32.0 (65.3%)	103 (66.0%)
**LEQ later-life bonus (education)**			
Mean (SD)	1.53 (4.09)	2.61 (6.10)	1.88 (4.80)
Median [Min, Max]	0 [0, 23.0]	1.26 [0, 26.0]	0.550 [0, 26.0]
Missing	72.0 (67.3%)	32.0 (65.3%)	104 (66.7%)
**WTAR FSIQ**			
Mean (SD)	113 (5.30)	112 (6.28)	113 (5.61)
Median [Min, Max]	114 [92.0, 122.0]	114 [83.0, 118.0]	114 [83.0, 122.0]

*Means, medians, and standard deviations (SD) presented for continuous measures. Otherwise, sample size and missing (% of total) values reported. BDNF, brain-derived neurotrophic factor, FTE, full-time equivalent; LEQ, lifetime experience questionnaire; SD, standard deviation; THBP, Tasmanian Healthy Brain Project; WTAR FSIQ, Weschler Test of Adult Reading Full-Scale Intelligence Quotient*.

### Model Selection

Variables with *p*-values <0.10 were *BDNF* Val66Met, gender, and the two LEQ subscales of education and training in young adulthood and mid-life (Table 2). These variables, and age, were retained in the reported multiple linear regression model. Years of university study with the THBP, LEQ young adulthood general, midlife specific—occupation, midlife general and WTAR FSIQ were not significantly associated with serum BDNF.

**Table 2:** Linear regression coefficients with 95% confidence intervals for the associations between age, BDNF Val66Met genotype, gender, WTAR FSIQ, education and included LEQ variables on serum BDNF concentration log_e_(pg/ml).

Predictors	Model 1 BDNF in serum log(pg/ml)			Model 2 BDNF in serum log(pg/ml)		
	Estimates	95% CI (lower, upper)	*p*	Estimates	95% CI (lower, upper)	*p*
(Intercept)	11.109	10.161, 12.058	**<0.001**	10.397	9.903, 10.892	**<0.001**
Age (years)	−0.005	−0.012, 0.003	0.21	−0.008	−0.015, −0.001	**0.032**
BDNF Val 66 Met genotype: met+	0.084	−0.006, 0.174	0.066	0.08	−0.009, 0.169	0.077
Gender: Male	−0.098	−0.195, −0.002	**0.046**	−0.109	−0.203, −0.015	**0.023**
WTAR FSIQ	−0.006	−0.014, 0.002	0.173
FTE Years of university	0.01	−0.024, 0.043	0.566
Study (THBP intervention)						
LEQ young adulthood specific (education and training)	0.009	0.003, 0.015	**0.004**	0.007	0.001, 0.012	**0.022**
LEQ young adulthood general	−0.001	−0.010, 0.008	0.902
LEQ midlife	−0.008	−0.019, 0.003	0.147
Specific (occupation)						
LEQ midlife general	−0.007	−0.017, 0.003	0.188
LEQ midlife bonus (education and training)	−0.006	−0.011, −0.001	**0.029**	−0.007	−0.012, −0.001	**0.013**
Observations	144			144		
R^2^/R^2^ adjusted	0.188/0.127			0.146/0.115		

*Bold values indicate p-values were considered significant (*p* < .05). BDNF, brain-derived neurotrophic factor; CI, confidence interval; FTE, full-time equivalent; LEQ, lifetime experience questionnaire; THBP, Tasmanian Healthy Brain Project; WTAR FSIQ, Weschler Test of Adult Reading Full-Scale Intelligence Quotient*.

### Inferential Statistics

**Figure 1:**
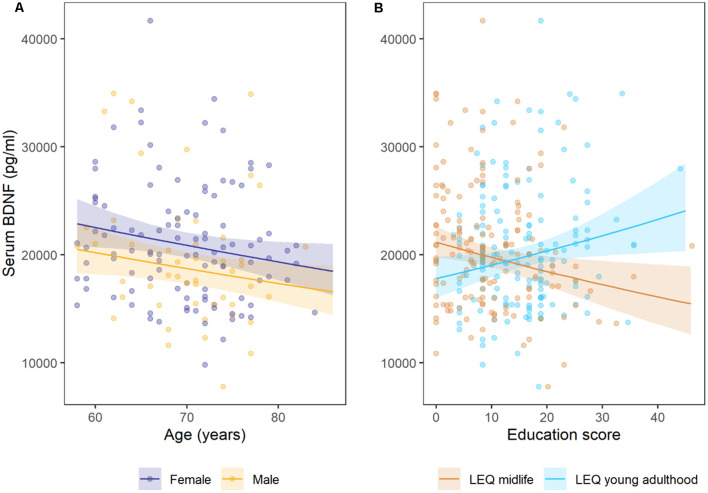
Estimated marginal mean trends for **(A)** age stratified by gender, **(B)** lifetime of experiences questionnaire (LEQ) midlife education and LEQ young adulthood education. Points are observed data, shaded regions represent 95% confidence intervals. Females had small, but significantly higher mean (log) serum BDNF than males, and concentrations decreased with each year of age (in adults older than 58 years of age). Cross-sectional serum BDNF concentrations increased with higher young adulthood education and training scores, but decreased with higher mid-life education and training scores on the LEQ.

Age was significantly associated with a −0.008 [95%CI −0.015, −0.001] decrease in log(pg/ml) serum BDNF concentrations per year of age (*p* = 0.032; $\eta_{p}^{2}$ = 0.036; Table 2). Males had −0.109 [−0.203, −0.015] lower log(pg/ml) serum BDNF concentrations than females (*p* = 0.023; $\eta_{p}^{2}$ = 0.022). Figure 1A illustrates the estimated marginal mean age trends stratified by gender. Participants who carried at least one Met allele had 0.08 [−0.009, 0.169] log(pg/ml) higher serum BDNF than Val homozygotes, but this difference was not significant (*p* = 0.077; $\eta_{p}^{2}$ = 0.025).

Education and training subscales were significantly associated with serum BDNF concentrations, but the direction of these associations differed for young adulthood and mid-life subscales (Figure 1B). Education and training in young adulthood (LEQ young adulthood specific—education) was associated with a 0.007 [.001, 0.012] increase in log(pg/ml) serum BDNF per unit on the subscale, while education and training in mid-life (LEQ midlife bonus—education) was associated with a −0.007 [−0.012, −0.001] decrease in log(pg/ml) serum BDNF (*p* = 0.022 and *p* = 0.013 for young adulthood and mid-life, respectively). Although significant, both effect sizes were small ($\eta_{p}^{2}$ = 0.044 for mid-life and $\eta_{p}^{2}$ = 0.037 for young adulthood, Figure 1). Model 2 explained 11.5% of variance (adjusted *R*^2^).

Since a correlation between education and training at different life stages would result in biased estimates, we fitted covariate-adjusted models for young adulthood and mid-life education and training variables separately and obtained the same coefficients and confidence intervals (to 3 decimal places) as the model with both variables included. We observed no significant correlation between education and training in young adulthood (LEQ young adulthood specific—education) vs. mid-life (LEQ midlife bonus—education, Pearson’s *r* = −0.03, *p* = 0.744).

## Discussion

We present a cross-sectional investigation of serum BDNF levels in healthy, older Australian adult participants of the THBP, a longitudinal study of the effects of a complex cognitive stimulation in the form of university-level education. Our findings indicate increasing age, lower cognitive stimulation in young adulthood, and higher cognitive stimulation in mid-life are associated with lower serum BDNF levels in healthy adults aged between 50 and 76 years. Whilst we may have predicted the positive association with early-life cognitive stimulation (Weinstein et al., 2014; Novkovic et al., 2015) and reduction in serum BDNF with aging (Erickson et al., 2010; Shimada et al., 2014), the lower concentration of serum BDNF in people with higher cognitive stimulation in mid-life was surprising. A number of explanations seem possible, and it may be useful to challenge some of the assumptions we have about BDNF and its presence in the periphery.

It is broadly accepted that higher BDNF level in the blood is a beneficial state, reflecting greater brain levels (Rasmussen et al., 2009). This aligns with lower serum BDNF levels throughout aging, as BDNF levels are associated with the decline in neural processing and function with increasing age (Shimada et al., 2014). Previous literature has indicated that lower serum BDNF levels were related to the development of mild cognitive impairment (Shimada et al., 2014), AD (Ng et al., 2019) and higher brain BDNF levels are related to slower rates of cognitive decline (Buchman et al., 2016). However, Laske et al. (2006) have shown that people in the early stages of probable AD had higher levels of serum BDNF than both cognitively normal people and people with more severe stage AD. Therefore, the relationship between serum BDNF levels and dementia is not clear and may be dependent on cognitive health and vary across normal and pathological cognitive aging trajectories.

Lower BDNF levels in the periphery may represent several scenarios, ranging from decreased production in the brain, reduced neurogenesis, reduced activity-dependent plasticity, less transport to the vasculature, greater uptake by neural cells and/or greater efficiency in the use of this factor. We currently lack a specific neural correlate of cognitive reserve, but it is a capacity that can be built through lifetime experience and provides functional compensation for the accumulation of pathology (Stern, 2002). Furthermore, as BDNF is released following cognitive stimulation (Novkovic et al., 2015), it may be that people with higher cognitive reserve find tasks of daily living less cognitively demanding than people with lower cognitive reserve, and thus release less BDNF into the serum. In the current study of healthy adults, we observed that higher levels of education in young adulthood and lower levels of education in mid-life were both significantly associated with an increase in serum BDNF. Although our results are cross-sectional, this indicates the relationship between cognitive stimulation and circulating pools of BDNF may vary throughout the lifespan.

The current study did not find any significant difference in serum BDNF levels between *BDNF* Met+ and Met- participants but did find significantly lower BDNF levels in males compared to female participants. Previous research has shown disparities in BDNF serum levels between *BDNF* Met+ vs. Met- participants, with increased levels shown in a study of 114 healthy controls (Lang et al., 2009), decreased levels demonstrated in 122 participants (66 healthy controls; 56 major depressive disorder, Ozan et al., 2010) and no difference found in a group of 198 participants [99 healthy controls; 99 amnestic mild cognitive impairment (aMCI), Yu et al., 2008]. Given strong gene × environment interactions in aging, the impact of *BDNF* Met polymorphism on circulating serum BDNF may vary throughout an individual’s life and be mediated through other biopsychosocial factors that were outside the scope of this study. Similarly, there has been a disparity in the effect of gender on serum BDNF levels, with no difference between the genders (Lang et al., 2009) as well as both higher (Shimada et al., 2014) and lower (Ozan et al., 2010) levels in females reported. Our results align closely with those reported by Weinstein et al. (2014), where there was no effect of the Val66Met BDNF polymorphism on serum BDNF levels and that participants in the two lowest quintiles of serum BDNF levels were significantly older, more likely to be males and have an education of at least college degree or higher. Although our study was unable to investigate hormonal pathways, serum BDNF levels may be related to sex-specific hormones, as BDNF levels are associated with estrogen, progesterone, and testosterone levels and affected by menopausal changes in estrogen and hormone replacement therapy (Begliuomini et al., 2007; Chan and Ye, 2017). Furthermore, the rate of sex hormone changes over aging differs between males and females (Pluchino et al., 2013) and thus the age of participants and sample sizes of studies may explain the differing results reported.

We did not observe a significant relationship between estimated premorbid intelligence or participation in the university-level education intervention and serum BDNF levels. This supports a previous study in healthy older adults between the ages of 65 and 85 years which assessed acute changes in serum BDNF levels following physical activity, mindfulness practice, or cognitive training and found that only physical activity caused a significant increase in serum BDNF levels (Håkansson et al., 2017). Furthermore, a study of older adults with aMCI demonstrated that there was no significant change in serum BDNF levels following 12-weeks of either group- or home-based cognitive interventions (Jeong et al., 2016). However, serum BDNF levels did significantly correlate with improvements in cognition following the group- and home-based cognitive interventions (Jeong et al., 2016). As we did not include a measure of physical activity in our study, we are unable to determine any differences in activity across strata of engagement with the university study intervention or mid-life education. We speculate participants with lower levels of mid-life education may potentially be in more physically demanding occupations, thereby increasing their circulating levels of serum BDNF. It is a substantial limitation that we were unable to measure changes in serum BDNF levels pre- and post-intervention. Ongoing data collection in the THBP will allow us to investigate the relationship between activities that build cognitive reserve and changes in serum BDNF levels over time. Participants who undertook later-life university study displayed similar levels of previous education and were a similar mean age to those who did not, therefore we do not consider these results to be at high risk of bias by non-random, self-selection. We acknowledge the limitations in this study of a small sample size, which is inherent to longitudinal studies of this length and the healthy cohort bias, as participants of THBP display higher levels of education compared to the general population. We were also unable to adjust for other variables that may explain additional variance in serum BDNF levels, such as physical activity, diet, metabolic diseases, smoking and other substance use, social engagement, and stress. A strength of the current study was that the immunoassay used to assess serum BDNF levels is specific for the mature form of the BDNF protein and does not detect pro-BDNF, which is important because pro-BDNF and BDNF have differing effects on the brain (Carlino et al., 2013; Polacchini et al., 2015).

In conclusion, this study demonstrates for the first time, in healthy older adults, that the period of life in which education and training take place may moderate the association between education and training and serum BDNF levels in later life. The observed associations were opposed, independent and their estimated effect sizes were similar, suggesting that education at different stages of life may be associated with different cognitive and neural demands. Education may build networks in young adulthood, whilst preserving existing networks in mid-life. We conclude that education is important at different stages of life and should be accounted for as covariates when estimating associations between age-related health predictors and serum BDNF.

## Data Availability Statement

The data analyzed in this study is subject to the following licenses/restrictions: only de-identified data from participants who have consented to external research collaborations may be shared upon request. Requests to access these datasets should be directed to e.hill@utas.edu.au.

## Ethics Statement

The studies involving human participants were reviewed and approved by University of Tasmania Health and Medical Human Research Ethics Committees (H0018265 and H0016317). The participants provided their written informed consent to participate in this study.

## Author Contributions

JC and EH drafted the manuscript. AB conducted statistical analysis. JV and MS designed and supervised the study. All authors contributed to the article and approved the submitted version.

## Conflict of Interest

The authors declare that the research was conducted in the absence of any commercial or financial relationships that could be construed as a potential conflict of interest.

## Publisher’s Note

All claims expressed in this article are solely those of the authors and do not necessarily represent those of their affiliated organizations, or those of the publisher, the editors and the reviewers. Any product that may be evaluated in this article, or claim that may be made by its manufacturer, is not guaranteed or endorsed by the publisher.
